# Activation studies with amino acids and amines of a β-carbonic anhydrase from *Mammaliicoccus (Staphylococcus) sciuri* previously annotated as *Staphylococcus aureus* (SauBCA) carbonic anhydrase

**DOI:** 10.1080/14756366.2022.2131780

**Published:** 2022-10-09

**Authors:** Andrea Angeli, Linda J Urbański, Clemente Capasso, Seppo Parkkila, Claudiu T. Supuran

**Affiliations:** a Dipartimento Neurofarba, Sezione di Scienze Farmaceutiche e Nutraceutiche, Università degli Studi di Firenze, Sesto Fiorentino (Florence), Italy; b Faculty of Medicine and Health Technology, Tampere University, Tampere, Finland; c Department of Biology, Agriculture and Food Sciences, Institute of Biosciences and Bioresources, Napoli, Italy; d Fimlab Ltd, Tampere University Hospital, Tampere, Finland

**Keywords:** Staphylococcaceae, carbonic anhydrase, activator, amine/amino acid, *Mammaliicoccus (Staphylococcus) sciuri*

## Abstract

A β-carbonic anhydrase (CA, EC 4.2.1.1) previously annotated to be present in the genome of *Staphylococcus aureus*, SauBCA, has been shown to belong to another pathogenic bacterium, *Mammaliicoccus (Staphylococcus) sciuri*. This enzyme, MscCA, has been investigated for its activation with a series of natural and synthetic amino acid and amines, comparing the results with those obtained for the ortholog enzyme from *Escherichia coli*, EcoCAβ. The best MscCA activators were D-His, L- and D-DOPA, 4-(2-aminoethyl)-morpholine and L-Asn, which showed K_A_s of 0.12 − 0.89 µM. The least efficient activators were D-Tyr and L-Gln (K_A_s of 13.9 − 28.6 µM). The enzyme was also also inhibited by anions and sulphonamides, as described earlier. Endogenous CA activators may play a role in bacterial virulence and colonisation of the host which makes this research topic of great interest.

## 1. Introduction

Carbonic anhydrases (CAs, EC 4.2.1.1), the enzymes which catalyse the interconversion between CO_2_ and bicarbonate according to Equations (1) and (2), are widespread in all life kingdoms, including Bacteria^1–5^. Of the eight genetically distinct CA families known to date, at least four (α-, β-, γ- and ι-CAs) are present in these organisms, in which they play crucial roles related to metabolism, pH regulation, acclimation in different niches in which bacteria grow, but also pathogenesis and virulence in the case of pathogenic species^4–6^.

(1) $${\text{EZn}}^{2+}{\text{-OH}}^{-}+{\text{CO}}_{2}⇌{\text{EZn}}^{2+}{\text{-HCO}}_{3}^{-}\overset{{\text{+H}}_{\text{2}}\text{O}}{⇌}{\text{EZn}}^{\text{2+}}{\text{-OH}}_{\text{2}}+{\text{HCO}}_{\text{3}}^{\text{-}}$$

(2) $${\text{EZn}}^{2+}{\text{-OH}}_{2}⇌{\text{EZn}}^{2+}{\text{-OH}}^{-}+{\text{H}}^{+}\;\;\;\text{-rate determining step-}$$

Inhibition of CAs belonging to various classes and organisms has been exploited pharmacologically for various applications for the last decdes, mainly by targeting human CA (hCA) isoforms, of which 15 are presently known^7–11^. Many such isoforms are targets for diuretics, antiobesity, antiepileptic, antiglaucoma or antitumor agents^7–11^. Inhibition of such enzymes from pathogenic bacteria, fungi or protozoans was on the other hand proposed as a new approach to develop antiinfectives with novel mechanisms of action, devoid of the drug resistant problems of the currently used agents^5^^,^^11^. Thus, a large number of drug design studies of CA inhibitors (CAIs) targeting both mammalian and pathogenic CAs are constantly being reported, mainly based on the tail approach developed by one of our groups over the last two decades^12^.

On the other hand, activation studies of various classes of CAs have progressed slower compared to the inhibition studies. The CA activation mechanism was definitively demonstrated at the molecular level only in 1997 with the report of the first X-ray crystallographic adduct of a CA – activator complex, more precisely hCA II complexed with histamine^13^. Thus, Briganti et al.^13^ demonstrated that CA activators (CAAs) participate directly in the enzyme catalytic cycle, as shown schematically in Equation (3), binding in a different binding site compared to the classical sulphonamide inhibitors, i.e. at the entrance of the cavity^6^^,^^13^.

(3) $$\begin{array}{l} {\text{EZn}}^{\text{2}+}{\text{-OH}}_{\text{2}}+\text{A}⇌[{\text{EZn}}^{\text{2}+}{\text{-OH}}_{\text{2}}-\text{ A}]\;⇌[{\text{EZn}}^{\text{2}+}-{\text{HO}}^{-}-{\text{ AH}}^{+}]\;\\ ⇌{\text{EZn}}^{\text{2}+}-{\text{HO}}^{-}+{\text{AH}}^{+}\\ \;\;\;\;\;\;\;\;\;\;\;\;\;\;\;\;\;\;\;\;\;\;\;\;\;\;\;\;\;\;\;\;\;\;\;\;\;\;\;\;\;\;\;\;\;\;\;\;\;\;\text{enzyme }-\text{ activator complexes} \end{array}$$

Presently, a large number of activation studies of all hCAs are available with many classes of compounds, and several crystallographic and drug design studies were also reported^14–17^. Furthermore, CAAs may have pharmacological applications for memory therapy as well as for the treatment of cognitive disorders in need of effective therapies^18^. Athough this field is still in its infancy, crucial advances have been made over the last few years in understanding the connections between fear, extinction/social memory and CA activation/inhibition^17^^,^^18^.

Non-mammalian CAs activation, mainly described in fungal and bacterial pathogens started to be investigated only in the last years, in order to understand whether endo- or exogenic modulators of this enzymatic activity may interfere with virulence, metabolism or pathogenicity of these organisms^19–21^. Indeed, CAs from fungi such as *Malassezia globosa, Saccharomyces cerevisiae, Candida albicans, Cryptococcus neoformans*, etc., or bacteria such as *Vibrio cholerae, Mycobacterium tuberculosis*, *Francisella tularensis*, *Brucella suis, Escherichia coli*, etc., were recently investigated for their activation profiles with natural and synthetic amines and amino acid derivatives^19–21^.

Among the pathogens investigated ultimately for the presence of druggable CAs, was *Staphylococcus aureus*, a bacterium known for its virulence and easy development of drug resistance to a variety of clinically used antibiotics^2^. In 2016 we identified in the NCBI database a sequence annotated as encoding for a β-CA in the genome of *S. aureus*, which we cloned, characterised and showed to be susceptible to inhibition with sulphonamides and anions, two of the most investigated classes of CAIs^2^. This enzyme, denominated SauBCA, showed the typical behaviour of a bacterial β-CA, possessing a significant CO_2_ hydrase catalytic activity, similar to those of other such enzymes described earlier in *E. coli, M. tuberculosis*, *Salmonella enterica* (serovar *Typhimurium*), and many other pathogenic bacteria by us and other groups^1–5^. However, a recent reinvestigation of the database showed that the initial annotation was erroneous, and that the sequence thought to belong to the genome of *S. aureus*, was in fact from another species of this genus, *Staphylococcus sciuri*^22^. To make things even more complicated, recently *S. sciuri* has been moved to another taxon, *Mammaliicoccus sciuri*^23^*. Mammaliicoccus (Staphylococcus) sciuri*, is known as a Gram-positive, oxidase-positive, coagulase-negative member of these infectious bacteria, provoking disease in humans and animals (it was originally isolated from the squirrel)^22^. In fact, the taxonomy of the *Staphylococcaceae* family is rather complex, and as mentioned earlier, many genome annotations were inexact or were overlapping between various genetically similar species^23^. However, all these bacteria provoke diseases in humans and animals and show variable (usually high) degrees of resistance to clinically used antibiotics^22^.

**Figure 1.**
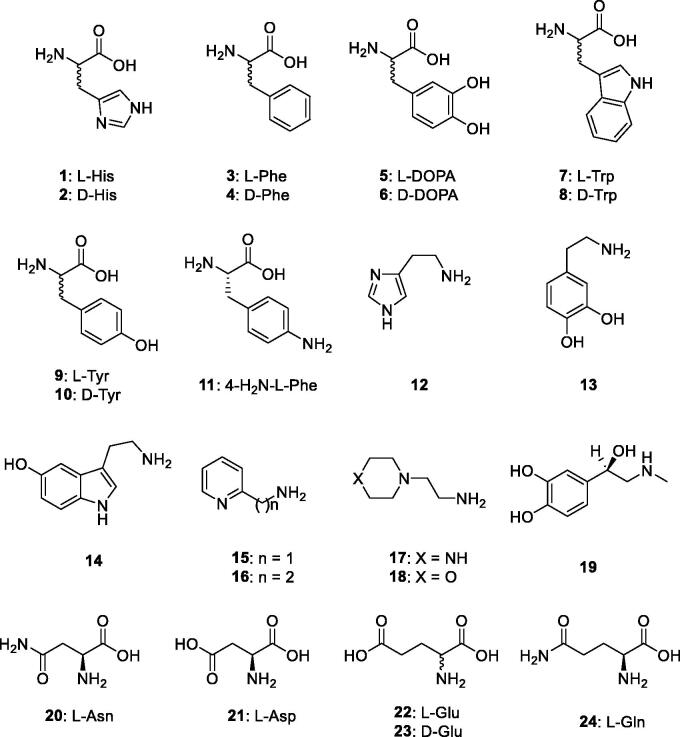
Amino acids and amines **1–24** investigated as CAAs in the current article.

Here we report an activation study of the β-CA previously known as SauBCA, and now renamed here as MscCA, with a series of amino acids and amines of types **1–24** (Figure 1) previously investigated as activators of other classes of CAs, including several bacterial such enzymes^19–21^. We also compare the obtained results with those for a similar β-class enzyme from the model orgnisms *Escherichia coli*, EcoCAβ, investigated earlier for its activation with the same class of compounds^21c^.

## 2. Materials and methods

### 2.1. Enzyme production and purification

The protocol described in ref.^2^ has been used to obtain purified recombinant MscCA. EcoCAβ was also obtained in-house as reported earlier^21c^.

### 2.2. Ca activity/activation measurements

An Sx.18Mv-R Applied Photophysics (Oxford, UK) stopped-flow instrument has been used to assay the catalytic activity of various CA isozymes for CO_2_ hydration reaction^24^. Phenol red (at a concentration of 0.2 mM) was used as indicator, working at the absorbance maximum of 557 nm, with 10 mM Hepes (pH 7.5, for α-CAs)^14–16^ or TRIS (pH 8.3, for β-CAs)^19–21^ as buffers, 0.1 M NaClO_4_ (for maintaining constant ionic strength), following the CA-catalyzed CO_2_ hydration reaction for a period of 10 s at 25 °C. The CO_2_ concentrations ranged from 1.7 to 17 mM for the determination of the kinetic parameters and inhibition constants. For each activator at least six traces of the initial 5–10% of the reaction have been used for determining the initial velocity. The uncatalyzed rates were determined in the same manner and subtracted from the total observed rates. Stock solutions of activators (at 0.1 mM) were prepared in distilled-deionized water and dilutions up to 1 nM were made thereafter with the assay buffer. Enzyme and activator solutions were pre-incubated together for 15 min prior to assay, in order to allow for the formation of the enzyme–activator complexes. The activation constant (*K_A_*), defined similarly with the inhibition constant K_I_, can be obtained by considering the classical Michaelis–Menten equation (Equation 4), which has been fitted by non-linear least squares by using PRISM 3:

(4) $$\text{v }={\text{v}}_{\text{max}}/\left\{\text{1}+\left({\text{K}}_{\text{M}}/\left[\text{S}\right]\right)\left(\text{1}+{\left[\text{A}\right]}_{\text{f}}/{\text{K}}_{\text{A}}\right)\right\}$$

where [*A*]*_f_* is the free concentration of activator.

Working at substrate concentrations considerably lower than *K_M_* ([*S*] ≪*K_M_*), and considering that [*A*]*_f_* can be represented in the form of the total concentration of the enzyme ([*E*]*_t_*) and activator ([*A*]*_t_*), the obtained competitive steady-state equation for determining the activation constant is given by Equation (5):

(5) $$\text{v}={\text{v}}_{0}.{\text{K}}_{\text{A}}/\{{\text{K}}_{\text{A}}+{\left({\left[\text{A}\right]}_{\text{t}}-0.\text{5}\{\left({\left[\text{A}\right]}_{\text{t}}+{\left[\text{E}\right]}_{\text{t}}+{\text{K}}_{\text{A}}\right)-{\left({\left[\text{A}\right]}_{\text{t}}+{\left[\text{E}\right]}_{\text{t}}+{\text{K}}_{\text{A}}\right)}^{\text{2}}-\text{4}{\left[\text{A}\right]}_{\text{t}}.{\left[\text{E}\right]}_{\text{t}}\right)}^{\text{1}/\text{2}}\}\}$$

where *v_0_* represents the initial velocity of the enzyme-catalyzed reaction in the absence of activator^19–21^. Enzyme concentrations in the assay system were of 7.6 − 12.8 nM.

### 2.3. Reagents

Amines and amino acid derivatives **1–24** were obtained in the highest purity that was available commercially from Sigma-Aldrich (Milan, Italy).

## 3. Results and discussion

The catalytic activity of MscCA is significant for the physiologic reaction, i.e. hydration of CO_2_ to bicarbonate, with a *k*_cat_ of 1.46 × 10^5^ s^−1^ and a Michaelis-Menten constant *K_M_* of 5.7 mM, these kinetic parameters being comparable to those of other α- or β-CAs investigated earlier^14^^,^^21^ (Table 1). The data in Table 1 also indicates that the presence of L-Trp as an activator does not change the *K_M_* for either of the two enzymes belonging to the α-class (hCA I/II) as well as for EcoCAβ and MscCA, a situation also observed for all CA classes for which CA activators have been investigated so far^13–17^^,^^19–21^. In fact, as proven by kinetic and crystallographic data^13–18^, the activator binds in a different region of the active site than the site of substrate binding. Thus, the activator does not influence K_M_ but has an effect only on *k*_cat_. Indeed, a 10 µM concentration of L-Trp leads to a 7.5-fold enhancement of the kinetic constant of MscCA compared to the same parameter in the absence of the activator (Table 1). For hCA I and II, the enhancement of the kinetic constant in the presence of L-Trp was rather modest, as these enzymes have a weaker affinity for this activator (Table 1). On the other hand, L-Trp has a low micromolar affinity for MscCA which explains its effective activating effect on this bacterial enzyme.

**Table 1.** Activation of human carbonic anhydrase (hCA) isozymes I, II, EcoCAβ and MscCA with L-Trp, at 25 °C, for the CO_2_ hydration reaction^25^.

	*k*_cat_*	K_M_*	(*k*_cat_)_L-Trp_**	K_A_*** (µM)
Isozyme	(s^−1^)	(mM)	(s^−1^)	L-Trp
hCA I^a^	2.0 × 10^5^	4.0	3.4 × 10^5^	44.0
hCA II^a^	1.4 × 10^6^	9.3	4.9 × 10^6^	27.0
EcoCAβ^b^	5.3 × 10^5^	12.9	1.8 × 10^6^	18.3
MscCA^c^	1.46 × 10^5^	5.7	1.10 × 10^6^	1.02

*Observed catalytic rate without activator. *K_M_* values in the presence and the absence of activators were the same for the various CAs (data not shown), **Observed catalytic rate in the presence of 10 µM activator; ***The activation constant (*K_A_*) for each enzyme was obtained by fitting the observed catalytic enhancements as a function of the activator concentration. All data are mean from at least three determinations by a stopped-flow, CO_2_ hydrase method^24^. Standard errors were in the range of 5–10% of the reported values (not shown). ^a^Human recombinant isozymes, from ref.^14^; ^b^Bacterial recombinant enzyme, from ref.^21^c, ^c^This work.

Thus, we proceeded with the investigation of activators **1–24** (Figure 1) belonging to the amino acid and amine chemotypes for understanding their ability to activate MscCA as well as the structure-activity relationship profiles. In Table 2, the activation constants of these compounds against the target enzyme MscCA as well as hCA II and II (α-CA enzymes) and EcoCAβ (a bacterial β-CA) are shown, for comparative reasons. The following SAR was observed for the activation of MscCA:

**Table 2.** Activation constants of hCA I, hCA II and the bacterial enzymes EcoCAβ (*E. coli*) and MscCA with amino acids and amines **1–24**, by a stopped-flow CO_2_ hydrase assay^24^.

No.	Compound	*K_A_* (µM)*			
		hCA I^a^	hCA II^a^	EcoCAβ^b^	MscCA^c^
**1**	L-His	0.03	10.9	36.0	5.24
**2**	D-His	0.09	43	23.7	0.47
**3**	L-Phe	0.07	0.013	12.0	1.25
**4**	D-Phe	86	0.035	15.4	8.62
**5**	L-DOPA	3.1	11.4	10.7	0.89
**6**	D-DOPA	4.9	7.8	3.14	0.40
**7**	L-Trp	44	27	18.3	1.02
**8**	D-Trp	41	12	11.5	3.45
**9**	L-Tyr	0.02	0.011	9.86	3.81
**10**	D-Tyr	0.04	0.013	17.9	13.9
**11**	4-H_2_N-L-Phe	0.24	0.15	7.34	0.73
**12**	Histamine	2.1	125	18.5	1.15
**13**	Dopamine	13.5	9.2	11.3	6.23
**14**	Serotonin	45	50	2.76	1.08
**15**	2-Pyridyl-methylamine	26	34	48.7	2.69
**16**	2-(2-Aminoethyl)pyridine	13	15	17.2	7.94
**17**	1-(2-Aminoethyl)-piperazine	7.4	2.3	14.1	3.52
**18**	4-(2-Aminoethyl)-morpholine 0.14	0.19	17.4	0.12	
**19**	L-Adrenaline	0.09	96.0	9.15	5.26
**20**	L-Asn	11.3	>100	49.5	0.88
**21**	L-Asp	5.20	>100	18.9	4.67
**22**	L-Glu	6.43	>100	18.0	3.75
**23**	D-Glu	10.7	>100	11.4	4.93
**24**	L-Gln	>100	>50	49.2	28.6

*Mean from three determinations by a stopped-flow, CO_2_ hydrase method^25^. Standard errors were in the range of 5–10% of the reported values (data not shown).

^a^Human recombinant isozymes, from ref.^14^; ^b^Bacterial recombinant enzyme, ref.^21^; ^c^Bacterial recombinant enzyme, this work.

- All investigated amines and amin acids showed activating effects against MscCA, with K_A_s ranging between 0.12 and 28.6 µM. It has been demonstrated earlier that the activator binds at the entrance of the CA active site (for α-class CAs^6^^,^^13–17^) and participates in the rate determining step of the catalytic cycle, the shuttling of the protons from the zinc coordinated water molecule to the reaction medium. In this way the nucleophilic metal hydroxide species of the enzyme is formed, which enhances the overall catalytic process^6^^,^^13–17^. Although no X-ray crystal structures of β-CA – activator complexes are known to date, we hypothesise that the activation mechanism is similar for all CA classes. This is also the reason why the CAAs possess protonatable moieties of the amino, imidazole and other heterocycles, or even carboxylate type^17^, all of them present also in comounds **1–24** investigated here.
- The most effective activators were D-His, 4-amino-L-Phe, L-and D-DOPA, 4-(2-Aminoethyl)-morpholine and L-Asn, which showed *K*_A_s of 0.12–0.89 µM. These derivatives belong to three different chemotypes: aromatic amino acids based on the His/Phe chemotype (**3, 5, 6** and **11**); heterocylic amines incorporating 2-aminoethyl side chains (**18**) and aliphatic dicaroxylic amino acid derivative (**20**). However, other investigated compounds structurally similar to these derivatives showed weaker CA activating effects, proving that the molecular recognition between the MscCA active site and the modulator is governed by many factors, and that small structural changes in the activator molecule leads to drastically different activating effects (Table 2). For example D-DOPA is an effective MscCA activator (K_A_ of 0.40 µM) whereas the structurally related D-Tyr and D-Phe (with one and no phenolic OH moieties, respectively) showed weaker such properties, with K_A_s of 13.9 and 8.62 µM, respectively. The same differences can be observed between the structurally related amines **17** and **18**, which differ only by the endocyclic X group, with the morpholine derivative **18** being 29.3-times a better activator compared to the piperazine **17**.
- Most of the investigated activatrs were effective, low micromolar activators of MscCA (*K*_A_s of 1.02–8.62 µM) – Table 2. They include compounds **1, 3, 4, 7–9, 12–17, 19, 21–23**, belonging both to the amino acid and amine series. There seem to be no preference for L- or D-amino acids, since in some cases the D-enantiomer was a better activator, whereas in other cases the L-enantiomer showed more effective activating effects. Furthermore, these activators, as the ones discussed above, belong to heterogeneous chemotypes, making the SAR rather difficult to dissect. What is important on the other fact is that many diverse structural motifs incorporating proton shuttling moieties of the amino, heterocylic or carbocylate type show these effects.
- The least effective activators against MscAC were D-Tyr and L-Gln, with *K*_A_s of 13.9–28.6 µM).
- The activation profile of MscCA is very different from that of other bacterial β-CAs, as the E. coli enzyme showed in Table 2, as well as the human isoforms hCA I and II.

## 4. Conclusions

The β-CA from *M. sciuri*, previously considered to be present in the genome of *S. aureus*, is effectively activated by amines and amino acids. Furthermore, as described earlier, this enzyme is also inhibited by anions and sulphonamides^2^. Recently, Götz’s group^25^ performed a thorough analysis regarding the presence of CAs in the genome of *S. aureus* and related species, expressing a rather critical vision regarding our earlier work on SauBCA^2^ and bacterial CAs in general^3^. It is true that we did not investigate in detail whether the *S. aureus* genome sequences present in the NCBI database are all correct, as this is not our main research interest. However, the experiments and statements in which the N-cyanosulphonamide S-0859 is considered as a selective inhibitor of sodium-bicarbonate cotransporters by Götz’s group in order to definitey demonstrate the absence of CAs in this bacterium^25^ are inconclusive, since N-cyanosulfonamides also act as rather effective CAIs^26^^,^^27^. Whether CAs are present only in some members of the *Staphylococcaceae* and not in others, is of course highly relevant, but it should be noted that bacteria may encode also for ι-CAs^3^, which were not searched for in the above-mentioned study^25^. What is more relevant according to us, is the fact that our study and the preceding ones^2^, although performed on an enzyme thought to belong to *S. aureus* but which is actually *M. sciuri*, may bring to attention druggable targets which may lead to antibiotics with a novel mechanism of action. In fact, several groups showed that inhibition of bacterial CAs represents an effective and innovative way for fighting drug resistant bacteria^4^^,^^5^, with all the scepticism from groups as the one mentioned above that these enzymes could be considered antiinfective drug targets. As far as we know, resistance to sulphonamide CAIs has not been registered for any of the investigated bacterial species, although this phenomenon is erroneously mentioned in ref. ^25^
